# Soil-transmitted helminths associated with BMI in schoolchildren of Chakdara, Lower Dir, Pakistan

**DOI:** 10.5455/javar.2025.l983

**Published:** 2025-12-25

**Authors:** Wali Khan, Ateeq Ullah, Majed H. Wakid, Tabana Iman, Zubia Masood, Tanzeela Yousaf, Patricio R. De los Rios-Escalante, Mashael Abdullah Aldamigh, Yousef Abdal Jalil Fadladdin

**Affiliations:** 1 Department of Zoology, University of Malakand, Chakdara, Pakistan; 2 Department of Zoology, Hazara University, Mansehra, Pakistan; 3 Department of Medical Laboratory Sciences, Faculty of Applied Medical Sciences, King Abdulaziz University, Jeddah, Saudi Arabia; 4 Special Infectious Agents Unit, King Fahd Medical Research Center, King Abdulaziz University, Jeddah, Saudi Arabia; 5 Department of Zoology, Sardar Bahadur Khan Women’s University, Quetta, Pakistan; 6 Department of Zoology, Women University, Swabi, Pakistan; 7 Departamento de Ciencias Biológicas y Químicas, Facultad de Recursos Naturales, Universidad Católica de Temuco, Temuco, Chile; 8 Núcleo de Estudios Ambientales, Universidad Católica de Temuco, Temuco, Chile; 9 Department of Biology, College of Science, Majmaah University, Al-Majmaah, Saudi Arabia; 10 Department of Biological Sciences, Faculty of Science, King Abdulaziz University, Jeddah, Saudi Arabia

**Keywords:** Intestinal parasite, school children, soil-transmitted helminths, BMI

## Abstract

**Objective:**

This study aimed to determine the association between soil-transmitted helminths and body mass index (BMI) among the school children of Chakdara, Lower Dir, Pakistan.

**Materials and Methods:**

Students aged 5–15 years participated in this study. Stool specimens were collected from 130 students between August 2020 and September 2021 and examined both macroscopically and microscopically. The height and weight were measured, then classified as normal BMI or underweight BMI according to the World Health Organization range criteria.

**Results:**

The overall prevalence was noted as 38.5%, with 35.9% of males and 43.9% of females being infected. Roundworms (56%) were the most prevalent, followed by hookworms (28%) and whipworms (16%). Students with 5–8 years of experience presented the highest prevalence rate of 41.2%, followed by those with 9–12 years (33.3%) and 13–15 years (40%). A total of 30% of children had a normal BMI, while 70% were underweight. Of the 38.5% of infected children, 26% had a normal BMI, while 74% were underweight. Lack of handwashing with soap, lack of footwear, and low family income were identified as significant risk factors (*p*-value < 0.05) for helminth infection, while other factors, such as family size and access to a toilet at home, showed no significant association (*p*-value = 0.05). The association between soil-transmitted helminths (STH) infection and underweight BMI (*p*-value = 0.20), even though a sizable portion of students possessed BMIs, heights, and weights below standard reference ranges.

**Conclusion:**

The present study concludes that underweight is a risk factor for STH infection, reflecting poor hygiene standards and malnutrition in children. To reduce infection rates, these children must adopt a better diet and practice better personal, environmental, and hygiene habits.

## Introduction

By definition, a parasite is an organism that lives in or on another organism for food and shelter; it may or may not cause disease, but it harms the host in any case. This includes an enormous range of species, ranging from protozoa (single-celled) to multicellular worms (helminths). Nematodes are parasitic helminths that cause illnesses in the human gastrointestinal tract after ingestion of the infective eggs or skin penetration by L3 larvae. According to the World Health Organization (WHO), the most common species of soil-transmitted helminths (STH) that infect humans are *Ascaris lumbricoides* (roundworms), *Trichuris trichiura* (whipworms), and hookworm (*Necator americanus* and *Ancylostoma duodenale*) [1]. Strongyloides stercoralis is among the STH, but among the most neglected tropical diseases, and has recently received increased attention in accordance with the WHO 2030 global target [2,3].

Anemia, vitamin A deficiency, underdeveloped growth, starvation, intestinal blockage, and eosinophilic pneumonia are all possible outcomes of these parasite infections [4]. Intestinal worms are projected to infect 3.5 billion individuals, with 1.47 billion people infected with *A*.* lumbricoides*, 1.3 billion with hookworm*,* and 1.05 billion people suffering with *T*.* trichiura*. [5]. Schoolchildren are especially vulnerable to parasitic diseases. Patients experience malnutrition and growth issues due to parasitic roundworms consuming nutrients from their digestive tracts. Children living in filthy conditions, with poor sanitation and little knowledge of worm infections, are at risk of contracting them and will only be able to receive a proper education when they are in good mental and physical health. In areas with poor sanitation, *A*. *lumbricoides* and *T*. *trichiura* eggs contaminate the soil. Transmission to humans occurs mainly with soil contamination of hands or ingestion of contaminated foods or drinks containing mature larvated (embryonated) eggs. In the case of hookworm, humans become infected through skin penetration (transcutaneous) with L3 larvae, typically via bare feet in both species, and via the oral route in the case of *A*. *duodenale* [4]. Infection with these parasites is common throughout tropical and subtropical regions, with the highest rates in Sub-Saharan Africa, the Americas, China, and East Asia. According to the global burden of STH, Asia accounts for about 70% of infections [6].

Pakistanis one of the countries victimized by intestinal parasites. According to previous studies conducted in various locations of Pakistan, substantial incidence rates exist for drug addicts (22.8% [7]), dog parasites (26.8% [8]), education departments (64.8% [9]), medical students (59.8% [10]), shepherds (36.8%[11])*,* intestinal helminth infection 12.4 % [12]), and intestinal protozoal infection (28.8% [7]). Intestinal parasites are a leading cause of death in emerging nations like Bangladesh, India, Pakistan, and others [13]. The infection of parasites is widespread in all stages of people, but children are more susceptible [14]. It is estimated that about 4 billion schoolchildren are expected to be infected with STH [15]. Teenage girls and childbearing women are commonly infected with hookworms [5]. Intestinal infections are one of the challenging issues that cause morbidity amongst children; therefore, control efforts should be focused on this group. The high frequency of STH is linked to poverty, inadequate health services, poor sanitation, lack of access to safe drinking water, family size, child health, and parental literacy [16,17].

According to the WHO, helminth parasites afflict 2 billion people, with over two-thirds of them infected with one type of intestinal parasite [18]. Several intestinal parasitic disorders, such as amoebiasis and giardiasis (caused by intestinal protozoans), as well as soil-transmitted helminthiasis, have been classified among neglected tropical diseases. These infections are characterized by nausea, intestinal discomfort, and constipation. The WHO had advised that parasitic infestations be effectively integrated into a multi-disease control strategy that included tuberculosis, malaria, and HIV/AIDS [19]. The WHO-recommended medicines are Albendazole (400 mg) and Mebendazole (500 mg). These drugs are given to national ministries of health in all endemic countries through the WHO for the treatment of all school-aged children [2].

A child’s nutritional state is a significant predictor of their general health, and growth is the most reliable worldwide measure of a child’s well-being [20]. The school-age years are the busiest during childhood [21]. The risk factors that are frequently known to cause abnormal growth patterns in children are inadequate dietary intake, unsanitary surroundings, and recurrent parasitic infectious diseases [20]. Climatic conditions, poor sanitation, economic status, lack of access to clean drinking water, malnutrition, and cultural traditions are all associated with intestinal parasitic infections [22,23].

STH is endemic in the northern regions of Khyber Pakhtunkhwa (KPK), with the highest prevalence in the Swat district (37%). In Pakistan, most of the southern parts have a very low rate of infection, with the prominent exclusion of the urban area (Karachi), where infection frequency exceeds 20%. World scientists and public health officials, including those in Pakistan, have paid little attention to STH until now. Therefore, efforts should be made to protect our environment from contamination with these life-threatening parasites and to reduce the spread of infectious diseases and prevalent undernutrition. The current research aimed to determine the association between STH and body mass index (BMI) among schoolchildren.

## Materials and Methods

### Ethical approval

This study was approved in its presented form by the Departmental Ethical Review Committee (DERC), including the chairman, supervisor, and external examiner of the Department of Zoology (Ref. No. UoM/Zool/BS/2017-21; date:5 October, 2021).

### Study area

**Figure 1.**
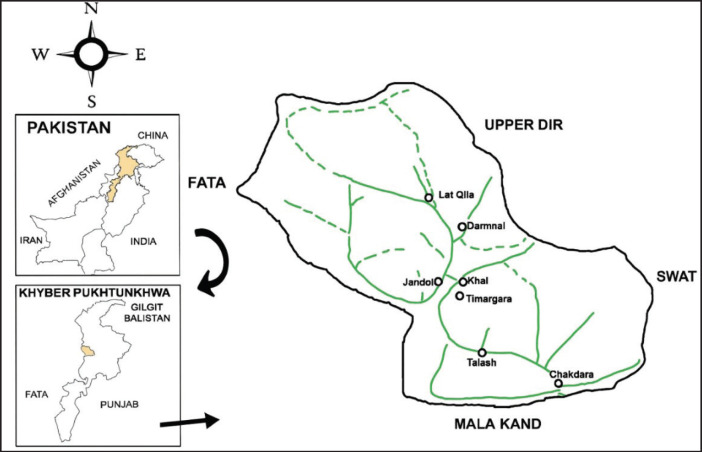
Map of Lower Dir, KPK, Pakistan.

**Figure 2.**
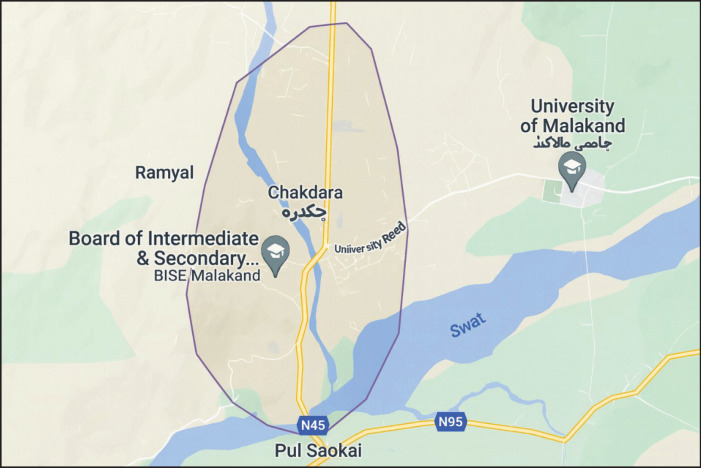
Map of the study area.

This cross-sectional study was conducted at Chakdara school, Lower Dir, KPK, Pakistan, from August 26, 2020, to September 14, 2021. The study site is located in the northern area of Malakand, near the River Swat, in a strategic position near the entrance to the Swat district and at the entrance to Dir (L). The temperature ranges from 26.5°C to 38°C. The population of the district is 1,435,917, with the majority residing in rural areas. The primary language spoken in the district is Pashto (Figs. 1 and 2).

### Sample collection

Before the study initiation, a meeting was held with the principal of the relevant school to discuss the study’s significance. A few days before the study began, during a parent-teacher meeting, parents were presented with details of the study, including anthropometric measurements, stool sample collection, and the child’s vital data. A questionnaire was prepared to record each participant’s information.

In this cross-sectional study, 150 labeled plastic containers with lids and instructions for collecting stool samples were distributed among schoolchildren in Chakdara, Lower Dir, Pakistan. One hundred thirty students, including both male and female participants, participated. The samples collected were preserved in a 10% formalin-saline solution for further investigation.

### Body mass index calculation

The children who gave their assent had their anthropometric data measured, which included their height in meters and weight in kilograms. The WHO standard formula was used to calculate the index, where BMI = weight (kg) / [height (m)]². A BMI range between 18.5 and 24.9 is considered “normal BMI,” while less than 18.5 is considered “underweight BMI” [24].

### Laboratory examination

Collected stool samples were examined macroscopically and microscopically as previously described [25,26]. Macroscopic examination included color, consistency, and the presence of adult worms. Microscopic examination of stool samples was performed using a light microscope in a well-established diagnostic parasitology laboratory to detect any diagnostic stage of the STH.

## Results and Discussion

**Figure 3.**
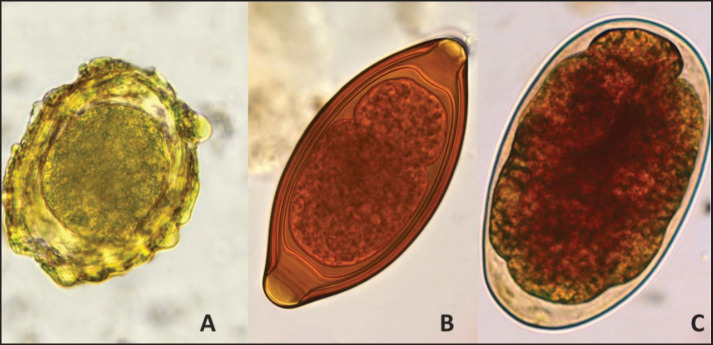
Detected parasites:(A) *Ascaris lumbricoides* egg; (B) *Trichuris trichiura* egg; (D) Hookworm egg.

As shown in Tables 1 and 2, this study included 130 students aged 5–15 years, of whom 89 (68.5%) were males with an infection rate of 35.9%, and 41 (31.5%) were females with an infection rate of 43.9%. The overall prevalence was 38.5% (50/130). The age group of 5–8 years showed the highest prevalence (41.2%), while the prevalence was also observed in the 9–12-year-old age group (33.3%). Among the 50 infected cases, *A*. *lumbricoides* (*n* = 28, 56%) was the most common intestinal helminth detected, followed by hookworm (*n* = 14, 28%), and *T*. *trichiura* was the least common (*n* = 8, 16%). No cases were infected with *S*. *stercoralis* (Tables 1,2, and Fig. 3).

**Table 1.** The prevalence of STH infections and schoolchildren’s gender.

Gender	Number	Infected cases, *N* (%)			
		*Ascaris lumbricoides*	Hookworm	*Trichuris trichiura*	Total
Male	89	18 (56.2)	9 (28.1)	5 (15.6)	32 (35.9)
Female	41	10 (55.5)	5 (27.7)	3 (16.6)	18 (43.9)
Total	130	28 (56)	14 (28)	8 (16)	50 (38.5)

**Table 2.** The prevalence of STH infections and schoolchildren’s age.

Age group (years)	Number	Infected cases, *N* (%)			
		*Ascaris lumbricoides*	Hookworm	*Trichuris trichiura*	Total
5–8	63	15 (57.6)	8 (30.7)	3 (11.5)	26 (41.3)
9–12	42	8 (57.1)	4 (28.5)	2 (14.2)	14 (33.3)
13–15	25	5 (50)	2 (20)	3 (30)	10 (40)
Total	130	28 (56)	14 (28)	8 (16)	50 (38.5)

Normal and underweight BMIs are displayed in Table 3, along with the age groups and infectivity status of the children. Infected children had lower body weight and height than uninfected schoolchildren, resulting in a lower BMI in infected children compared with uninfected children. However, this difference was not statistically significant (*p*-value = 0.20). The considerable hazard issues of STH infection were also analyzed in Table 4. Chi-square test analysis revealed a significant association between the prevalence of disease and risk factors, as well as other socio-economic characteristics, including handwashing with soap, footwear habits, and family income (*p*-value < 0.05). The analysis revealed that walking barefoot is a significant factor strongly associated with STH infection (*p*-value = 0.0002).

**Table 3.** BMI and infectious status in relation to each other.

Age group (years)	Normal BMI			Underweight BMI		
	Uninfected *N* (%)	Infected *N* (%)	Total *N* (%)	Uninfected *N* (%)	Infected *N* (%)	Total *N* (%)
5–8	12 (46.1)	6 (46.1)	18 (46.1)	31 (57.4)	21 (56.7)	52 (57.1)
9–12	8 (30.8)	5 (38.5)	13 (33.3)	16 (29.6)	12 (32.4)	28 (30.8)
13–15	6 (23)	2 (15.4)	8 (20.5)	7 (13)	4 (10.8)	11 (12)
Total	26 (66.7)	13 (33.3)	39 (100)	54 (59.3)	37 (40.7)	91 (100)
Chi-square 0.40				3.21		
*p-*value	0.8			0.20		

*p* < 0.05

**Table 4.** Socioeconomic traits and STH infection.

Variable	Category	*N* (%)	Infected, *n* (%)	Chi-square ( *χ* ²)	*p* -value
Handwashing with soap	Yes	102 (78.5)	27 (54)	10.6	0.001
	No	28 (21.5)	23 (46)		
	Total	130	50		
Footwear habit	Yes	93 (71.5)	21 (42)	13.5	0.0002
	No	37 (28.5)	29 (58)		
	Total	130	50		
Presence of toilet at home	Yes	115 (88.5)	42 (84)	0.64	0.42
	No	15 (11.5)	8 (16)		
	Total	130	50		
Number of family members	< 4	3 (2.3)	1 (2)	0.56	0.44
	4–8	48 (36.9)	17 (34)		
	9–12	37 (28.5)	12 (24)		
	>12	42 (32.3)	20 (40)		
	Total	130	50		
Monthly income (Rs)	<10,000	9 (6.9)	7 (14)	10.7	0.013
	10,000–15,000	17 (13)	15 (30)		
	15,000–20,000	40 (30.8)	10 (20)		
	>20,000	64 (49.2)	18 (36)		
	Total	130	50		

One of the primary health issues that prevents school-age children from achieving their optimal performance is worm infestation, which compromises immunity, mental health, and BMI. Children are most susceptible to parasitic infections due to their weakened immune systems, frequent contact with dust and contaminated objects, and lack of awareness of the importance of hygiene and health standards [27,28]. The present research explores the knowledge regarding STH infection among schoolchildren from Chakdara, Lower Dir, KPK, Pakistan, without prior use of anthelmintic drugs by the studied individuals.

Comparing this study with the findings of [29] in Abeokuta, Ogun state, Nigeria, the prevalence of STH infection from the school in Chakdara, Lower Dir, Pakistan, was high (38.5%). This high prevalence could be attributed to open-field defecation, walking barefoot, a lack of handwashing with soap after using the toilet, and failure to clean fruits before eating. The high frequency of STH infection is also associated with risk factors such as unplanned urbanization, poorly designed sewerage systems, and poor sanitation [23].

The high incidence of STH infection was consistent with the findings of [11], which involved Swat shepherds, both male and female. Research conducted in India also revealed a high incidence in Gujarat, ranging from 60% to 70% [30]. On the other hand, a study in Edo State showed a low frequency of 0.7%, in contrast to the current prevalence of intestinal parasite infection [31]. This low incidence was also observed in the Ugandan study [32].

In the current study, *A*. *lumbricoides*, *T*. *trichiura,* and hookworm were detected as STH. The most common parasite was *A*. *lumbricoides*, known to be one of the main parasites found in communities with inadequate sanitation standards [28,33,34]. According to our study, *A*. *lumbricoides* (56%) was the most common STH, which is consistent with other studies [35,36] where prevalences of 27.1% and 39.8% were reported, respectively. A higher prevalence of 68.3% was also reported [37]. Previous studies have reported prevalences of 2.17%, 4.3%, and 12.5% for *A*. *lumbricoides* [38-40].

In this study, hookworm and *T*. *trichiura* presented a prevalence of 28% and 16%, respectively. Hookworm was the second most prevalent STH in the current study, compared to [33] (12.7%), but differs from the results of [7], who reported a lower prevalence of 4.46% for this nematode. The low prevalence of hookworms in the current study contrasts with [39], which reported a high prevalence of hookworms (56.2%). The same survey of intestinal infections among school-aged children in Nepal reported a prevalence of 2.89% for *T. trichiura* [39], which is consistent with the findings of the present study. This differential prevalence of STH may be attributed to several factors, including malnutrition, poor sanitation, lack of access to safe drinking water, inadequate child health, poverty, low parental literacy rates, large family sizes, and insufficient health services [23].

In the current investigation, STH infections were more prevalent in female (43.9%) than in male children (35.9%), which is consistent with the report value of [11], who suggested that the prevalence rate of STH was sex determinant, in line with [29] in Abeokuta, Ogun. However, a study by [39] revealed that intestinal helminth parasite infections were more common in male than female children. Another investigation involving schoolchildren in Nigeria found no discernible difference in the prevalence of parasites between the two sexes [41,42.

In the current study, the prevalence was higher among children aged 5–8 (41.2%) compared to other age groups (9–12 and 13–15 years), reaching 33.3% and 40%, respectively. Previous research conducted in Nigeria supports this high prevalence in this population group [43]. This may be due to the development of their immunity against parasites at this stage of their life. On the other hand, intestinal parasite infection is not age-dependent, as noted in [44].

Anthropometric measurement is now an effective means of assessing the nutritional status of populations, particularly children in developing countries, as nutritional status is the most reliable measure of overall child well-being worldwide [45,46]. This study found a significant correlation (*p*-value > 0.05) between intestinal parasite infection and malnutrition, including children’s nutritional status.

The current study found that a significant proportion of children in the study area were malnourished, and children with normal BMI (>18.5–24.9) had lower parasitic infection rates than underweight children (BMI < 18.5). Overall, a significant number of school-going children in the field area were severely affected by intestinal worms (*p*-value < 0.05). Among the infected children, 30% had a normal BMI and 70% had a low BMI. Among uninfected children, 26% had normal BMI and 74% had low BMI, which agrees with previous studies [47–51]. More than 50% of the infected children present with low BMI during their anthropometric measurements, a finding consistent with a previous study [52]. The current work found no significant association between STH infection and underweight BMI (*p*-value = 0.20); therefore, aspects such as food accessibility, household clutter security, and communicable infection might be expected to be more important in influencing nutritional status and thus STH prevalence among schoolchildren.

In our study, chi-square analysis revealed a significant association between the given risk factors and STH infection (*p*-value < 0.05), with the strongest association reported for not using footwear (*p*-value = 0.0002). Similarly, [17,53] found that not wearing footwear was associated with STH infection, whereas another study contradicted this observation [54]. The current study observed that some of the infected subjects had no proper toilet at their household, but this was a non-significant factor in contracting STH infection (*p*-value = 0.42). A previous study observed that children who used to defecate outdoors had a high prevalence of STH infection due to contaminated soil containing helminth eggs [53]. Other risk factors, such as handwashing with soap and family income, were also significant (*p*-values = 0.001, 0.004, and 0.013, respectively). However, the absence of a toilet at home and large family size did not show any significant association with the prevalence of STH infection.

## Conclusion

This study revealed that STH remains prevalent in the study area and poses a significant health problem among schoolchildren. This calls for improvements in children’s lifestyles and nutritional quality. Reducing this high prevalence requires considerable attention to personal, community, and environmental hygiene, which will have a positive impact on children’s physical development and overall well-being. Based on the findings of this investigation, it was determined to give the study area the best possible chance of surviving these infections, against which we have no adequate defense, by launching an awareness campaign on geohelminth infection, transmission, and prevention among schoolchildren.

## List of abbreviations

BMI, body mass index; HIV, human immunodeficiency virus; KPK, Khyber Pakhtunkhwa; STH, Soil-transmitted helminths; WHO, World Health Organization.
